# A pilot study analyzing the influence of the material and the size of the orthodontic archwire on the level of pain and anxiety in adult patients in treatment with brackets: A prospective triple-blind randomized clinical trial

**DOI:** 10.4317/jced.61428

**Published:** 2024-04-01

**Authors:** Raquel Marzal, Alberto Albaladejo, Adrián Curto

**Affiliations:** 1 Postgraduate Student in Orthodontics, Faculty of Medicine, University of Salamanca, Spain. Alfonso X el Sabio Avenue. 37007. Salamanca. Spain; 2 DDS, Full Professor in Orthodontics, Faculty of Medicine, University of Salamanca, Spain. Alfonso X el Sabio Avenue. 37007. Salamanca. Spain; 3 DDS, Professor in Pediatric Dentistry, Faculty of Medicine, University of Salamanca. Alfonso X el Sabio Avenue. 37007. Salamanca. Spain

## Abstract

**Background:**

The objective was to investigate the influence of the material and dimensions of the orthodontic archwire on the pain and anxiety in adult patients in orthodontic treatment with brackets.

**Material and Methods:**

A randomized prospective triple-blind clinical pilot study was conducted at the Dental Clinic of the University of Salamanca. The study sample comprised 30 adult patients who started orthodontic treatment with brackets. This sample was divided into two groups: the NiTi group (n=15) and Cu-NiTi group (n=15). Pain was analyzed with a visual analogue scale (VAS) and anxiety with the State–Trait Anxiety Inventory (STAI). Anxiety was assessed at the start of treatment (T0) and after one month (T1). Pain was analyzed at the start of treatment (T0), at different time points at the start (T01), and after 4 (T02), 24 (T03), and 48 hours (T04); these measurements were also recorded one month after starting orthodontic treatment (T11, T12, T13, and T14).

**Results:**

The mean age of patients was 31.3 (± 6.05) years old. The highest level of pain, at the beginning of treatment, was observed after 48 hours (5.57 ± 1.72) and at one month after starting treatment at 24 hours (5.13 ± 1.89), with no significant differences between the two groups. When analyzing anxiety, no differences were observed between groups; the anxiety levels were higher one month after starting treatment compared to the start. Regarding the correlation between pain and anxiety, the NiTi group showed a greater direct relationship (<0.05) between these two variables at the start of treatment in the anxiety trait in relation to pain at T02 and T03 and after a month in T12, T13, and T14.

**Conclusions:**

In the sample studied, there was no significant influence of the size or material of the orthodontic archwire on pain and anxiety levels.

** Key words:**Orthodontics, Brackets, Archwire, Pain, Anxiety, NiTi, Cu-NiTi.

## Introduction

During orthodontic treatment, the forces applied to the teeth cause pressure on the periodontal ligament; this compression will result in the presence of ischemia and inflammation in this ligament. The biochemical processes that occur during the movement of the teeth in orthodontic treatment lead to patients experiencing pain and discomfort during treatment (1).

Pain, during the early stages of orthodontic treatment with fixed braces, is very prevalent. Pain can be considered as the most negative side effect related to orthodontic treatment (2). In the early stages of orthodontic treatment, pain can affect up to 90% of patients. The level of pain perceived by patients is subjective and varies between individuals. This problem often results in non-adherence to treatment by the orthodontic patient; it may even deter patients from initiating orthodontic treatment (3).

Dental anxiety refers to tension and the fear of dental treatment and is a common phenomenon in patients (4). The majority of patients who begin orthodontic treatment have dental anxiety, due to their malocclusion and the perception they have about it (5).

Pain is a factor that directly influences the dental anxiety described by patients (1,3,4). Pain and dental anxiety are influenced by different factors such as sex, age, emotional state, or the patient’s previous painful experiences. Psychological factors exert an important influence on pain perception (6).

Previous studies have evaluated the influence of the bracket system used (conventional brackets, self-ligating brackets, etc.) on the pain and anxiety of orthodontic patients, with contradictory results observed between different authors (7,8).

In recent years, new material compositions have been developed for use in orthodontic archwires, including copper–nickel–titanium (Cu-NiTi) wires. Various *in vitro* studies have been conducted to evaluate the performance of these new Cu-NiTi archwires (9); however, very few clinical studies have been published that address the possible influence of the orthodontic archwire material on pain levels (10).

The objective of this randomized clinical study was to analyze the influence of the size and material of the orthodontic archwire on the levels of pain and anxiety described by patients, during a follow-up period of one month. The null hypothesis was that the pain and anxiety levels of the treated patients would not be influenced by either the size or the material of the orthodontic archwire used.

## Material and Methods

-Study Design

This pilot prospective study was planned as a randomized triple-blind trial with two parallel arms and a 1:1 allocation ratio. The methods were not changed after trial initiation.

The study was approved by the Research Ethics Committee of the University of Salamanca (study reference number: 1073) and followed the guidelines set by the Declaration of Helsinki for human research and the CONSORT guidelines.

Participants were informed of the examination procedures and were assured of the confidentiality of the information collected. Before recruitment, signed consent was obtained from each participant. Consecutive patients who were to undergo orthodontic treatment with fixed appliances and who consented to the study attended the University of Salamanca Dental Clinic from October 2023 to January 2024.

Participants were randomly assigned to one of the two study groups (the nickel–titanium (NiTi) group or copper–NiTi (Cu-NiTi) group), according to an electronic randomization method using the Research Randomizer website (www.random.org/sequences/). This study was designed as a triple-blind study; neither the participants, nor the researcher, nor the person evaluating the data knew of the intervention assigned to each study group. The specialist who evaluated the data received them encoded.

-Eligibility Criteria for Participants

The inclusion criteria were as follows: adult patients (> 18 years); patients with permanent dentition (except for third molars); mandibular anterior irregularity index ranging from 4 to 10 mm; and patients who started orthodontic treatment with brackets and in whom the appliances could be cemented from the beginning of treatment in both arches.

The exclusion criteria were: patient history of previous orthodontic treatment; patients in need of orthodontic treatment including extractions; patients in need of orthodontic/surgical treatment; patients with craniofacial anomalies; patients with untreated caries; patients with untreated gingival and/or periodontal pathology; patients with symptoms or diagnosed temporomandibular joint pathology; patients on treatment with anti-inflammatory drugs, analgesics, anxiolytics and/or antidepressants; or pregnant patients.

-Interventions

Participants were bonded with 0.022-inch MBT prescription stainless steel brackets (VictoryTM, 3MUnitek, California, USA) in both arches. At the first appointment, the upper and lower brackets and the tubes of the first permanent molars were cemented. The archwire sequence was 0.014 inch NiTi (Ormco, California, USA) (NiTi group) or Cu-NiTi (Ormco, California, USA) (Cu-NiTi group) at baseline and 0.016 inch NiTi (Ormco, California, USA) (NiTi group) or Cu-NiTi (Ormco, California, USA) (Cu-NiTi group) one month after starting treatment. Type of engagement with the elastomeric ligature (Dentaurum GmbH & Co., KG, Ispringen, Germany) was the same for all patients. Both groups were treated similarly by the same orthodontist (A.C.). By using a single orthodontist, differences caused by the intervention being carried out by several specialists were eliminated.

**Figure 1:**
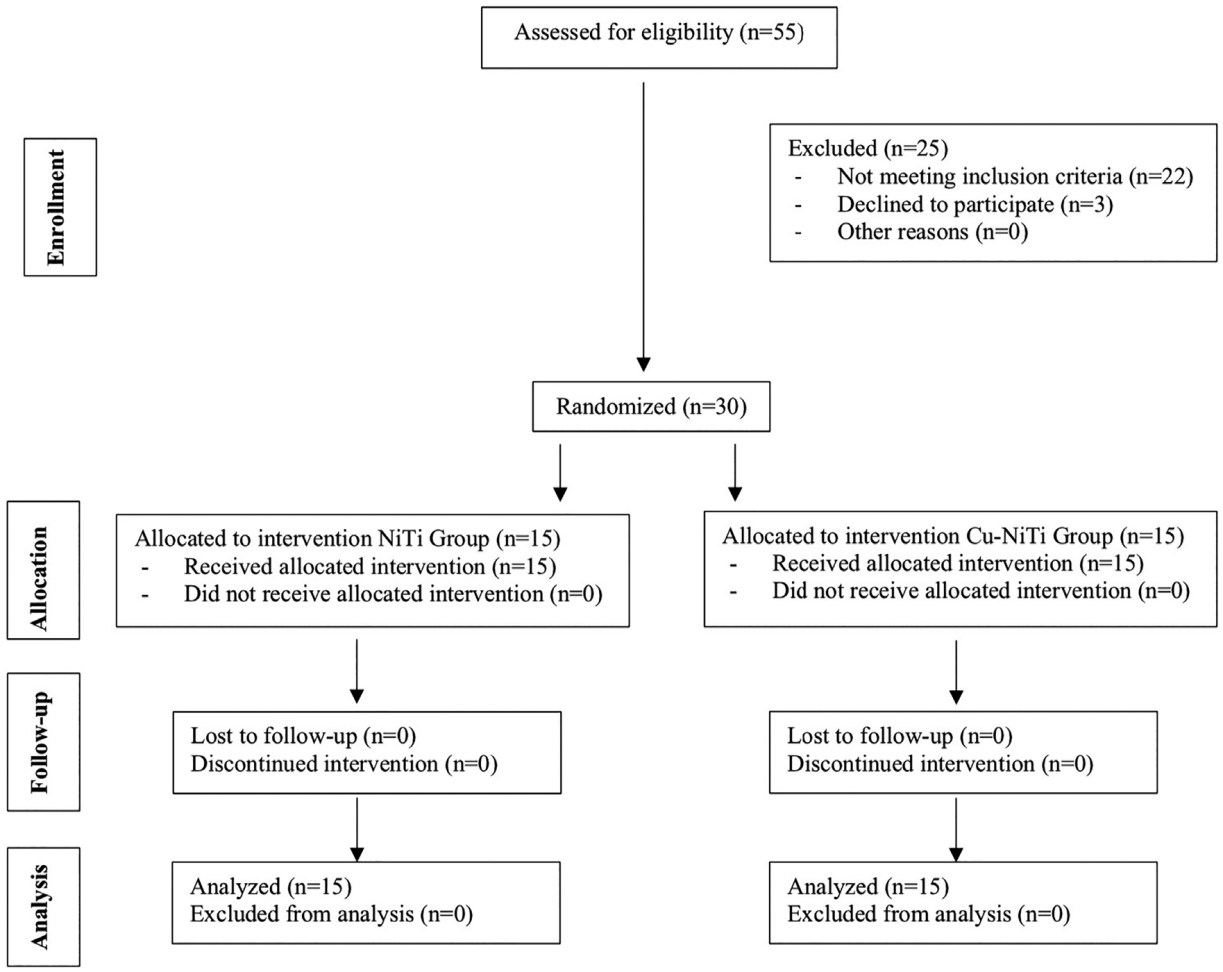
CONSORT diagram showing the flow of participant data during the trial.

A total of 55 patients were evaluated for inclusion in this study. According to the inclusion criteria, refusal to participate in this study, or other reasons, 25 patients were excluded. Finally, 30 patients were selected according to the randomization plan. The CONSORT diagram displaying the flow of this study is shown in Figure 1.

-Pain Analysis

A 100 mm visual analogue scale (VAS) was used to rate the level of pain experienced, with a score of 0 indicating “no pain” and a score of 10 indicating “maximum pain.” Patients had to describe their pain at baseline (T0) and one month after starting treatment (T1). At each moment of the evaluation, patients scored their pain on the visual analogue scale at baseline (T01 /T11), at 4 hours (T02/T12), at 24 hours (T03/ T13), and at 48 hours (T04/ T14) (11).

-Analysis of Anxiety

Anxiety was assessed using the State–Trait Anxiety Inventory (STAI). The STAI questionnaire is a 40-item Likert scale that assesses separate dimensions of anxiety as a state (items 1-20) and as a trait (items 21-40). Seventeen of these items must be recoded (inverse score) before calculating the total score of the questionnaire. This questionnaire was provided to patients at baseline (T0) and one month after starting treatment (T1). A score greater than 40 points is generally considered to be an indication of a high degree of anxiety (12).

-Statistical Analysis

Qualitative variables were analyzed with Tables of frequencies, percentages, Student’s t-test and the Chi-square test; quantitative variables were explored using the Shapiro–Wilk Test; the Mann–Whitney Test was used to contrast between group means; and the Spearman correlation coefficient was used to examine the association between numerical variables. In all statistical tests, statistical significance was considered when *p* < 0.05, while *p* < 0.01 indicated a high degree of statistical significance. The data were analyzed with the SPSS 28.0 software package.

## Results

-Baseline Data

The Student’s t-test and the Chi-square test confirmed that there were no statistically significant differences in the composition of the two groups. The total sample was 30 patients, with 15 participants in each study group, and with a mean age of 31.3 (± 6.05) years old. In relation to sex, 17 patients were men (56.7%), and 13 were women (43.3%) (Table 1).

**Table 1:**
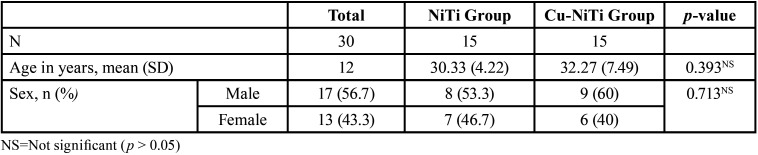
Baseline characteristics of patients in both groups.

-Analysis of Pain

When analyzing pain in the total sample, there were statistically significant differences (*p* < 0.01) when comparing pain scores at the start (T01) and at one month after starting treatment (T11). Patients described a higher level of pain at one month (1.47 ± 2.1) than at the baseline (1.4 ± 2.14). At the baseline, the highest pain score recorded in the VAS was at 48 hours (T04); however, one month after starting treatment, the highest level of pain was at 24 hours (T13) (Table 2).

**Table 2:**
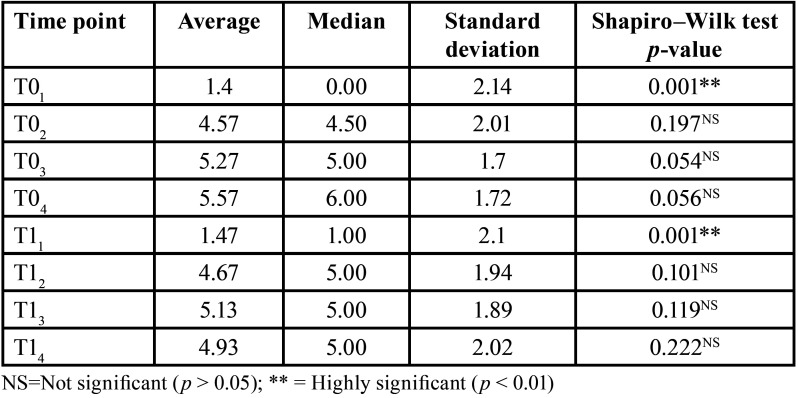
Pain analysis (VAS) in the total sample (n=30).

When analyzing the influence of age and sex on the level of pain in the analyzed sample, there was no statistically significant relationship (*p* > 0.05) between these two variables and the pain score in the VAS.

Pain was analyzed between the two treatment groups. The results indicated that, in general, there were no statistically significant differences, except at the baseline (T01). At this point, patients with the NiTi archwire described a higher level of pain (1.73 ± 1.53) compared to the Cu-NiTi group (1.07 ± 1.36). At this point, we must keep in mind that a 0.16 archwire was placed after one month. In both study groups, the highest pain scores at baseline (T0) were reported at 48 hours after cementing the brackets (T04); at one month after starting treatment, the highest level of pain was at 24 hours (T13). In both study groups, the lowest level of pain was at the start (T01 and T11) (Table 3).

**Table 3:**
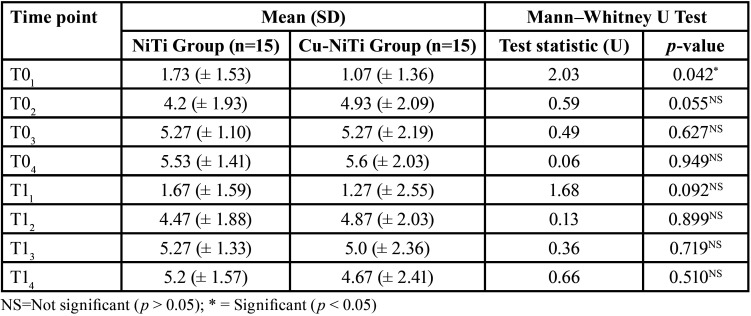
Comparison of pain (VAS) between the study groups.

-Analysis of Anxiety

In the total sample (n=30), there were statistically significant differences (*p* < 0.05) in anxiety measurements between the two study time points (T0 and T1). At both time points, the state anxiety level was higher than the trait anxiety level. The highest anxiety scores were described one month after starting treatment (T1) (Table 4).

**Table 4:**
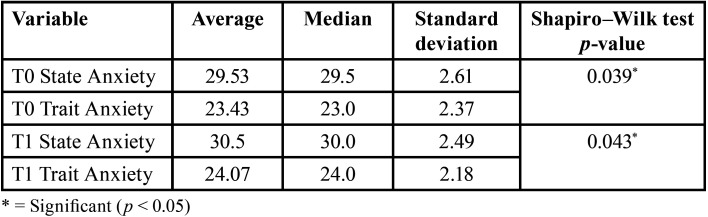
Analysis of anxiety (STAI questionnaire) in the total sample (n=30).

No significant differences were observed (*p* > 0.05) in the degree of anxiety with regard to the age or sex of the participants, according to the STAI questionnaire and in the sample studied.

When analyzing anxiety between the two study groups, there were no statistically significant differences between the two; therefore, we can conclude that, in the sample studied, neither the material nor the size of the orthodontic archwire influenced the participants’ anxiety levels. In both study groups, higher levels of anxiety were observed one month after starting treatment (T1) compared to the baseline (T0) (Table 5).

**Table 5:**
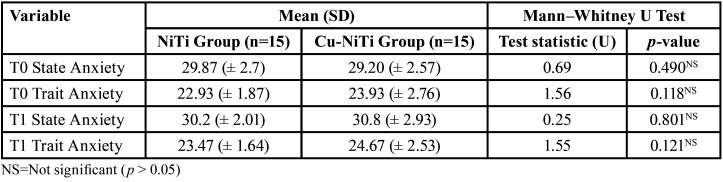
Comparison of anxiety (STAI) between the study groups.

-Correlation between Pain and Anxiety

We analyzed the correlation between the pain scores and the anxiety levels described by participants in the total sample. The only significant statistical difference was observed in the state anxiety levels at the baseline (T0) with respect to pain in the first 4 and 24 hours one month after starting treatment (T12 and T13) (*p* < 0.05). In this case, a significant degree of correlation was found, in that higher values of state anxiety were related to higher values in the perception of pain, especially after the first 4 hours to a month after starting orthodontic treatment (Table 6).

**Table 6:**
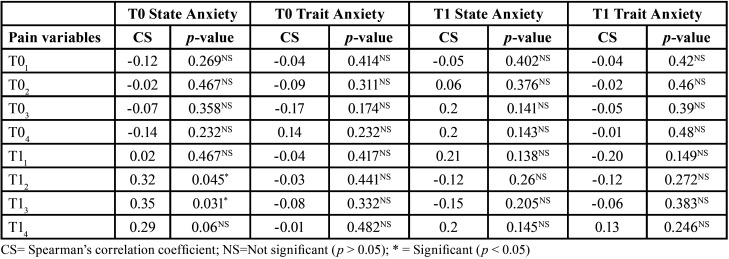
Association between the anxiety and pain variables in the total sample (n=30).

When analyzing the relationship between anxiety and pain in each group, for the most part, there was a relationship between these two variables in the NiTi group. In the NiTi group, a significant correlation was reported between trait anxiety and pain, one month after starting treatment, at T02, T03, T12, T13, andT14. In the Cu-NiTi group, a significant relationship between state anxiety and the level of pain was only found at the start of treatment, at T14. The higher the level of anxiety, the higher the level of pain observed in the study participants. In the NiTi group, a greater correlation between pain level and trait anxiety was reported, especially one month after starting Treatment ( Table 7, Table 8).

**Table 7:**
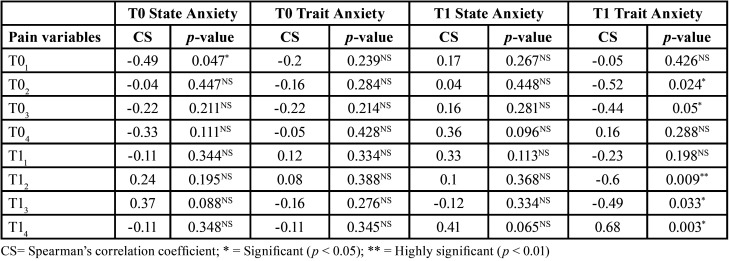
Association between the anxiety and pain variables in the NiTi archwire group (n=15).

**Table 8:**
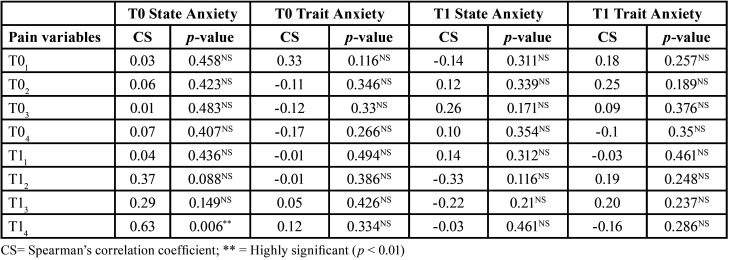
Association between the anxiety and pain variables in the Cu-NiTi archwire group (n=15).

## Discussion

The visual analogue scale has been widely used in various previous studies in the field of dentistry and orthodontics; it is a simple and objective tool to assess the level of pain described by patients (13). To determine the level of anxiety in patients undergoing orthodontic treatment, the STAI anxiety questionnaire has also been used in various previous studies (14,15). Pain is a complex sensation that varies from individual to individual, making it difficult to quantify in an objective way (2).

In this study, participants described their highest level of pain 48 hours after starting orthodontic treatment, and after 24 hours one month after starting treatment. The results described here are consistent with those reported in other previous studies, which describe that the peak of pain is reached in the first 24 (16) to 48 hours (2,17) after cementation and placement of the orthodontic archwire. In this study, we did not observe any statistically significant influence of age or sex on the pain levels described by the participants, which is consistent with the results reported by other authors (16).

We did observe that anxiety levels, according to the STAI questionnaire, were higher one month after starting treatment (T1) compared to the baseline (T0) in both study groups; however, no statistically significant differences were reported between the two groups in relation to anxiety. We did not observe a significant influence of the size of the orthodontic archwire on the patients’ anxiety level during the initial alignment phase. Aksoy *et al*., in 2019, analyzed the possible influence of the size of the orthodontic archwire on pain, anxiety, and salivary cortisol levels. These authors described higher levels of state anxiety (in the STAI questionnaire) at the start of treatment (42.10 ± 1.13) compared to the first month (38.80 ± 1.27) (18); these results are in contrast to those observed in our study. In our case, patients described a higher level of state anxiety at one month (30.5 ±2.49) compared to the start of treatment (29.53 ± 2.61).

In our study, there was no difference in anxiety levels between men and women, which coincides with the data reported by other authors (18-20).

The levels of anxiety in this study were directly related to the pain described by patients during their orthodontic treatment, especially in the NiTi group, at 4, 24, and 48 hours (*p* < 0.05) after orthodontic archwire insertion. This directly proportional relationship between pain and anxiety has also been reported by previous authors. It is reasonable to assume that higher levels of pain may produce higher levels of anxiety in patients (21-23).

The results of this study did not demonstrate a significant relationship of patients’ age and sex on their anxiety levels, and this is consistent with findings described by other authors (15), However, some studies do report a direct relationship between age and anxiety, such as Yavan (2021) who reported a positive correlation between age and the level of trait anxiety (*p* < 0.05) in patients undergoing orthodontic treatment with brackets; the older the patients, the higher the level of trait anxiety (24).

Reducing anxiety levels during orthodontic treatment is critical to increasing patient compliance and satisfaction; therefore, it is important to incorporate anxiety as an essential variable for treatment success (25,26).

As mentioned above, there are published studies that have analyzed the efficacy of Cu-NiTi archwires (9,27); however, these are *in vitro* studies. Azizi *et al*. analyzed the influence of orthodontic archwire material (NiTi versus Cu-NiTi) on pain in orthodontic patients. This author did not report any statistically significant differences in pain levels or duration between the two groups (10). In the case of our study, we only observed significant differences (*p* < 0.05) when comparing NiTi and Cu-NiTi at the beginning of treatment (T01). In this study, we observed higher levels of pain in the NiTi group (1.73 ± 1.53) compared to patients with Cu-NiTi archwires (1.07 ± 1.36).

Atik *et al*. compared the efficacy in teeth alignment by evaluating NiTi and Cu-NiTi archwires. In this study, there were no differences in the efficacy of the anterior maxillary alignment between the NiTi and Cu-NiTi archwires. The use of NiTi and Cu-NiTi archwires had similar effects on the changes produced in the intercanine and intermolar width (28).

-Strengths and limitations

Some of the strengths of the study were its randomized prospective triple-blind clinical study design with homogeneous study groups. The data obtained in this study can be used to inform orthodontic patients before starting their treatment. Providing information to patients prior to the start of their orthodontic treatment can improve patient compliance.

The current scientific literature is limited in the analysis of the pain and anxiety that patients describe during orthodontic treatment, when analyzing the influence of the material and the size of the archwire. The convenience of the analyzed sample, made up of patients recruited at the Dental Clinic of the University of Salamanca, may limit its representativeness and, therefore, prevent the generalization of these results.

The sample size and the follow-up period were limitations of this study. As a pilot study, a sample size of 30 participants was considered adequate to explore possible associations between the orthodontic archwire size and material in relation to the two study variables, pain and anxiety.

In future research, it will be necessary to extend the patients’ follow-up period, expand the sample size, and evaluate the effects of different orthodontic archwire sequences on the level of pain and anxiety perceived by patients.

## Conclusions

- There was no direct relationship between the material or the size of the orthodontic archwire and pain or anxiety in the sample studied.

- When analyzing pain, at the beginning of treatment, the highest score was described in the first 48 hours; one month after starting treatment, the highest level of pain was reported 24 hours later.

- Anxiety levels were higher one month after starting orthodontic treatment compared to the start. In both groups, state anxiety was greater than trait anxiety.

- In the population analyzed, neither sex nor age influenced anxiety levels or pain.
